# Participatory Disease Surveillance for the Early Detection of Cholera-Like Diarrheal Disease Outbreaks in Rural Villages in Malawi: Prospective Cohort Study

**DOI:** 10.2196/49539

**Published:** 2024-07-16

**Authors:** Mariana Gasparoto Pereira Valerio, Beverly Laher, John Phuka, Guilherme Lichand, Daniela Paolotti, Onicio Leal Neto

**Affiliations:** 1 Global Studies Institute University of Geneva Geneva Switzerland; 2 Kamuzu University of Health Sciences Lilongwe Malawi; 3 Graduate School of Education Stanford University Stanford, CA United States; 4 ISI Foundation Turin Italy; 5 Department of Epidemiology and Biostatistics Mel & Enid Zuckerman College of Public Health University of Arizona Tucson, AZ United States

**Keywords:** participatory surveillance, digital epidemiology, interactive voice response, cholera, public health, outbreak, cohort study, public health surveillance, health technology, digital surveillance

## Abstract

**Background:**

Cholera-like diarrheal disease (CLDD) outbreaks are complex and influenced by environmental factors, socioeconomic conditions, and population dynamics, leading to limitations in traditional surveillance methods. In Malawi, cholera is considered an endemic disease. Its epidemiological profile is characterized by seasonal patterns, often coinciding with the rainy season when contamination of water sources is more likely. However, the outbreak that began in March 2022 has extended to the dry season, with deaths reported in all 29 districts. It is considered the worst outbreak in the past 10 years.

**Objective:**

This study aims to evaluate the feasibility and outcomes of participatory surveillance (PS) using interactive voice response (IVR) technology for the early detection of CLDD outbreaks in Malawi.

**Methods:**

This longitudinal cohort study followed 740 households in rural settings in Malawi for 24 weeks. The survey tool was designed to have 10 symptom questions collected every week. The proxies’ rationale was related to exanthematic, ictero-hemorragica for endemic diseases or events, diarrhea and respiratory/targeting acute diseases or events, and diarrhea and respiratory/targeting seasonal diseases or events. This work will focus only on the CLDD as a proxy for gastroenteritis and cholera. In this study, CLDD was defined as cases where reports indicated diarrhea combined with either fever or vomiting/nausea.

**Results:**

During the study period, our data comprised 16,280 observations, with an average weekly participation rate of 35%. Maganga TA had the highest average of completed calls, at 144.83 (SD 10.587), while Ndindi TA had an average of 123.66 (SD 13.176) completed calls. Our findings demonstrate that this method might be effective in identifying CLDD with a notable and consistent signal captured over time (*R*^2^=0.681404). Participation rates were slightly higher at the beginning of the study and decreased over time, thanks to the sensitization activities rolled out at the CBCCs level. In terms of the attack rates for CLDD, we observed similar rates between Maganga TA and Ndindi TA, at 16% and 15%, respectively.

**Conclusions:**

PS has proven to be valuable for the early detection of epidemics. IVR technology is a promising approach for disease surveillance in rural villages in Africa, where access to health care and traditional disease surveillance methods may be limited. This study highlights the feasibility and potential of IVR technology for the timely and comprehensive reporting of disease incidence, symptoms, and behaviors in resource-limited settings.

## Introduction

Cholera-like diarrheal diseases (CLDDs) are a group of diarrheal illnesses that share similar pathogenesis and symptoms with cholera but are caused by agents other than the *Vibrio cholerae*, such as *Escherichia coli* and some strains of *Campylobacter*, *Yersinia*, *Aeromonas*, and other pathogens [1]. Typically, CLDDs are responsible for milder and shorter episodes of diarrhea than cholera, but a comprehensive clinical assessment, including laboratory testing, is essential for accurate diagnosis, especially when occurring in cholera-endemic areas like Malawi [1-3]. The epidemiological profile of CLDD is characterized by seasonal patterns, often coinciding with the rainy season when contamination of water sources is more likely [4-6]. Therefore, the global burden of diarrheal disease lies heavily in regions that face disruptive environmental and socioeconomic conditions that affect the water supply and sanitation infrastructure. Most of these regions are in Sub-Saharan Africa, where cross-border cholera outbreaks, lack of testing capacity, and surveillance system limitations add an extra layer of complexity to the situation [7-14]. On December 5, 2022, cholera was declared a public health emergency, and it has been considered the country’s worst outbreak of the past 10 years [15]. The rise in cases expected during the wet season has extended to the dry season, with deaths reported in all 29 districts since March 2022 [15,16].

The situation grew even more alarming after the flooding caused by Tropical Storm Ana and Cyclone Gombe in January and March 2022, respectively [17]. These phenomena led to population displacement and water, sanitation, and hygiene (WASH) infrastructure disruption, exacerbated by longstanding issues such as the inadequate preparedness for disease control, the absence of a reliable and geographically distributed early detection system, and shortages in the oral cholera vaccine and other treatments due to multiple outbreaks within the African region [2,5,15,17-19]. As of April 4, 2023, the World Health Organization (WHO) has reported 160,756 cases of suspected cholera in the African region with a case fatality ratio (CFR) of 2.1%. Malawi has been significantly affected, accounting for 35% (n=56,763) of the total cases and 52% (n=1722) of the total fatalities, demonstrating a consistently high CFR above 3% [16]. Unfortunately, the true burden of cholera is likely higher than the reported figures due to underreporting and lack of access to health care in many areas [7,8].

In line with the urgent need for comprehensive action, it is crucial to focus on viable strategies that can complement traditional epidemiological surveillance and foster community engagement [20]. In this field, participatory surveillance (PS) has proven to be valuable for the early detection of epidemics. This system engages the community in a bidirectional manner, capturing strategic data from the community, processing the acquired knowledge, and providing nearly real-time information back [21-23]. This approach involves the active participation of community members, health workers, and other stakeholders in the surveillance process, including reporting and monitoring of disease incidence, symptoms, and behaviors. PS has demonstrated applicability in different scenarios and diseases, such as influenza, cholera, COVID-19, Zika virus, and others [21,24-31]. One of the main advantages of PS is that it overcomes several challenges associated with traditional disease surveillance methods. PS empowers communities to take ownership of their health and well-being, allowing for more comprehensive and timelier case reporting [32,33].

PS using interactive voice response (IVR) technology is a promising approach for disease surveillance in rural villages in Africa for collecting timely and comprehensive data on disease incidence, symptoms, and behaviors in resource-limited settings [34,35]. Additionally, IVR technology is cost-effective and has the potential to improve disease surveillance and control efforts in low-income settings [35-37]. Furthermore, it is an easy-to-use technology that requires minimal training and can reach a wide range of people, including those in remote and rural areas, where access to health care and traditional disease surveillance methods may be limited.

This study aims to evaluate the feasibility and outcomes of low-cost, high-frequency, and high-quality data collection through PS for the early detection of CLDD outbreaks in Malawi, implementing a system to identify its early signals.

## Methods

### Overview

**Figure 1:**
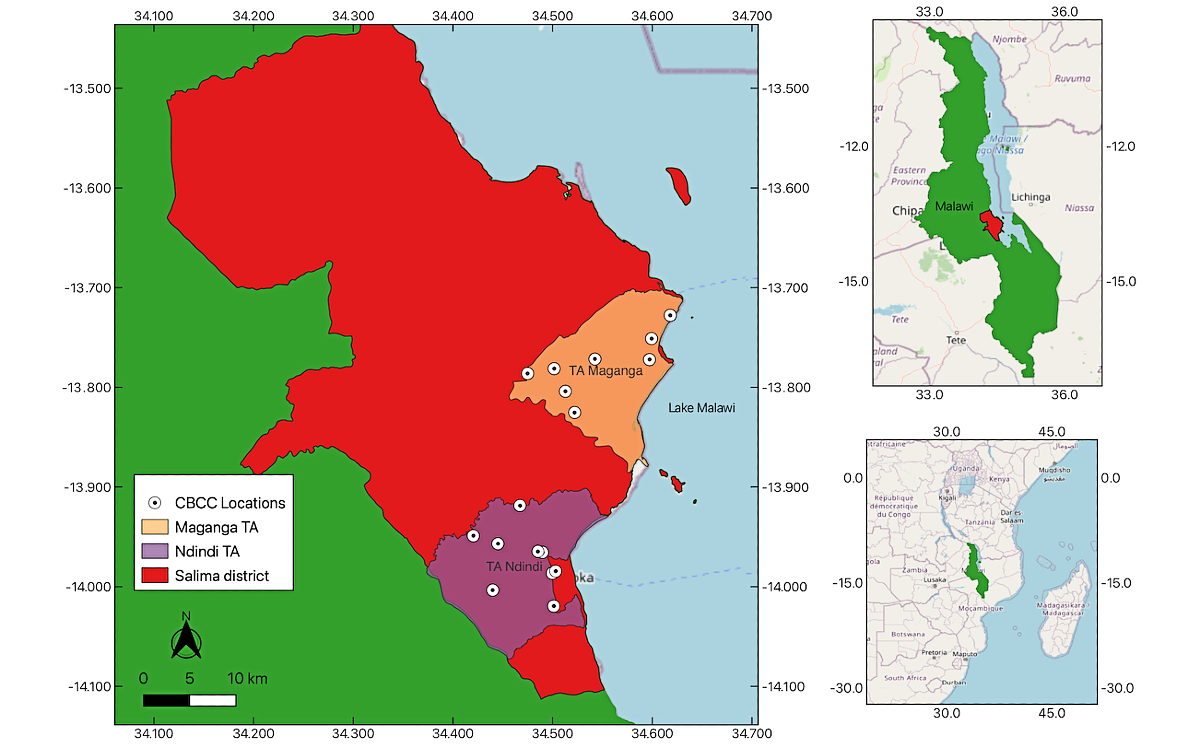
Study area highlighting the locations of the Salima district, Maganga Traditional Authority (TA), Ndindi TA, and the community-based childcare centers (CBCCs).

This longitudinal prospective cohort study followed 740 rural households in Malawi for up to 24 weeks. It was rolled out in 2 Traditional Authorities (TAs) located in Salima District, the central region of Malawi (Figure 1). The choice of the TAs took into consideration the broader context of a larger study—the Child Development Study, an initiative to leverage high-frequency data collection and novel technologies for understanding child development in low-income settings. Therefore, logistical considerations and resource availability guided the choice of these TAs as suitable candidates for inclusion.

A total of 4051 households were enrolled in the Child Development Study. From this total, a sample randomization was performed, selecting 740 households from both TA areas, representing 3743 household members, including 2393 children. For the purpose of this study, it is imperative to highlight the definition of a household in Malawi as “one or more persons, related or unrelated, who make common provisions for food and who regularly take their food from the same pot and/or share the same grain house (nkhokwe) or pool their incomes together for the purpose of purchasing food” [38].

The recruitment strategy was implemented by the Kamuzu University of Health Sciences (KUHES) local team, together with trained enumerators responsible for collecting the households’ phone numbers, conducting a baseline survey, and gathering metadata. All these interactions, including the sensitization campaigns promoted throughout the study, took place at the community-based childcare centers (CBCCs), as the inclusion criteria for the main study required at least 1 child attending a CBCC.

### Ethical Considerations

The College of Medicine Research and Ethics from Malawi (COMREC; reference no P_11_20_3202) and the Human Subjects Committee of the Faculty of Economics, Business Administration, and Information Technology at the University of Zurich (2018-046) approved this study. All participants signed the informed consent prior to enrollment and were compensated for their time spent reading and responding to messages in accordance with COMREC guidelines. Additionally, all phone costs were covered by the project.

### Data Collection via IVR Technology

The survey instrument was designed with 10 questions about symptoms to be answered weekly by the same household member over a period of 24 weeks (Table 1). Additionally, the design allowed all types of phones to be included, with participants required to simply type, press, or dial the numbers indicated by the voice message to respond with “yes” or “no.” The IVR was live from July 18, 2022, until January 8, 2023. Calls were conducted in Chichewa, the primary language spoken in Malawi. The survey content was pretested with in-country KUHES representatives to reduce events of loss in translation and misinterpretation. The survey asked whether anyone in the household had experienced specific symptoms in the past 7 days, including fever, headache, joint pain, vomiting/nausea, jaundice, chills, body ache, bleeding, and diarrhea. Additionally, participants were asked if anyone in the household had undergone a malaria test during the same period, with response options including “took a test with a negative result,” “did not take a test, and “took a test with a positive result.”

The proxies, and consequently, the questions used in the survey were selected based on the literature [39,40]. Special attention was given to designing questions capable of gathering comprehensive information while minimizing participant fatigue and enhancing adherence, as the same questions were repeated weekly. For this reason, the survey used proxies for malaria, rash, respiratory diseases, and CLDD. The rationale for these proxies was based on different disease characteristics: (1) exanthematic and ictero-hemorrhagic for endemic diseases or events and (2) diarrhea and respiratory for targeting both acute and seasonal conditions. This study specifically focused on reports related to CLDD, defined by the occurrence of diarrhea AND fever OR vomiting/nausea. Other proxies and their associated syndromes will be explored in future studies.

The household heads received calls from Monday to Friday, between 8 AM and 10 AM (a retry pattern of 2 calls with 2-hour intervals) and follow-up calls in the evening between 5:30 PM and 7:30 PM, running with this pattern throughout the week. An extract, transform, and load (ETL) process was performed weekly to feed data into a dashboard, allowing for near-real-time data visualization of the compiled results.

**Table 1:** List of questions and answers collected. Adjustments and translations were made in Chichewa, keeping the meaning of each symptom.

Question	Answer
Have you or anybody in your house experienced fever in the last 7 days?	If yes, dial 1; if, no dial 2.
Have you or anybody in your house experienced a headache in the last 7 days?	If yes, dial 1; if, no dial 2.
Have you or anybody in your house experienced joint pain in the last 7 days?	If yes, dial 1; if, no dial 2.
Have you or anybody in your house experienced vomiting/nausea in the last 7 days?	If yes, dial 1; if, no dial 2.
Have you or anybody in your house experienced jaundice in the last 7 days?	If yes, dial 1; if, no dial 2.
Have you or anybody in your house experienced chills in the last 7 days?	If yes, dial 1; if, no dial 2.
Have you or anybody in your house experienced body aches in the last 7 days?	If yes, dial 1; if, no dial 2.
Have you or anybody in your house experienced bleeding in the last 7 days?	If yes, dial 1; if, no dial 2.
Have you or anybody in your house experienced diarrhea in the last 7 days?	If yes, dial 1; if, no dial 2.
Did you or anybody in your house take a malaria test in the last 7 days?	If you took the test with a negative result, dial 1; If you did not take a test, dial 2; and if you took a test with a positive result, dial 3.

### Sensitization Campaigns and Participant Engagement

Sensitization campaigns were conducted to raise awareness among participants before the calls officially started. Additionally, during the development of the study, we organized meetings at each CBCC used as a reference point for the study (Figure 1). Based on the eligibility criteria, all households involved in the study had at least 1 child who regularly attended the CBCCs. To ensure widespread coverage and engagement, we conducted sensitization campaigns at the CBCC locations. This approach made it easier for participants to access the information and encouraged their active involvement. The campaigns were held at various times and on different days to maximize participation and reach as many people as possible. They were facilitated by an Assistant Environmental Health Officer, a social welfare representative, and the Association of Early Childhood Development Personnel in Malawi. These campaigns aimed to raise awareness and inform participants about the process of calls and interactions. They addressed any concerns or questions about technical issues related to using mobile phones in this specific context, reinforced that the repetitive calls were intentional and not spam or mistakes, and discussed the importance of preventive health measures. Table 2 describes the framework of the sensitization campaigns.

**Table 2:** Sensitization campaigns framework for engaging participants during the prospective cohort.

Description	Purpose
Demonstrative live call	To introduce the IVR^a^ system to potential participants by giving them a live demonstration of how it works. This allows participants to become familiar with the technology and understand how to use it.
Presentation explaining in simple words how the data would be collected and the relevance of the study.	To explain the purpose of the study and the importance of the data being collected. This helps participants understand how their participation will contribute to the success of the project and how it will benefit child development and health.
Clarification and reinforcement that the calls would come weekly from the same number, addressing the same questions.	To ensure that participants understand the frequency and consistency of the calls they will receive. This reinforces the importance of their participation and helps to build trust and rapport with the participants.
Presentation explaining the importance of preventive and early detection of outbreaks and empowering the participants as key collaborators to the success of such initiatives	To motivate and empower participants to take an active role in the project. By understanding the importance of their participation in preventing and detecting outbreaks, the participants are more likely to engage fully with the IVR system and provide accurate information.
Reinforcement about the possibility of calling back in case the participant missed a call.	To ensure that participants understand that they can still participate even if they miss a call. Because we provide clear instructions on this and reinforce that there is no cost involved, participants are more likely to remain engaged with the project.
Enquirement for the preferable time spot to receive the calls.	To allow participants to choose a convenient time for the weekly calls. By accommodating the schedules of participants, the project is more likely to receive accurate and consistent data.
Opening time for participant's questions and further requests for clarifications.	To provide participants with an opportunity to ask questions and seek further clarifications about the project. This helps to build trust and rapport with the participants and ensures that they are fully informed about the purpose and methodology of the study.

^a^IVR: interactive voice response.

### Statistical Analysis

We computed the household attack rate as an indication of how many households were at risk over the studied period. We assumed household reports as a proxy for the risk of presenting any of those symptoms related to CLDD. The attack rate is expressed as follows:



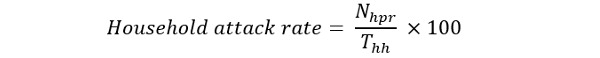



where *N_hpr_* is the household positive reports (number of true reports on the given proxy), and *T_hh_* is the total number of households in the study.

We used a polynomial generalized additive model (GAM) to analyze a time-series data set, given that the independent variable was time. The covariates were CLDD reports, diarrhea-like reports, fever-like reports, and vomiting/nausea-like reports, and they were chosen as nonparametric due to the uncertain relationship with the outcome.

The GAM is a flexible regression technique that allows for nonlinear relationships between the predictor variables and the response variable. To evaluate the performance of the polynomial GAM, we calculated *R*^2^, deviance explained, generalized cross-validation (GCV), and scale estimate. The *R*^2^ values ranged from 0 to 1, with higher values indicating a better fit of the model to the data. The following formula was used to estimate the coefficient of determination:



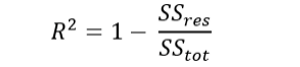



where *SS_res_* is the sum of squared residuals, and *SS_tot_* is the total sum of squares.

Deviance explained measures the reduction in deviance, which is a measure of the difference between the observed data and the fitted values, achieved by the model. Deviance-explained values range from 0 to 1, with higher values indicating a better fit of the model to the data. The formula is described as follows:



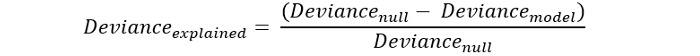



where *Deviance_null_* is the deviance of a null model with no predictors, and *Deviance_model_* is the deviance of the model being evaluated.

The GCV score is the average of the squared differences between the predicted values and the actual values. To estimate the GCV score, we used the following formula:



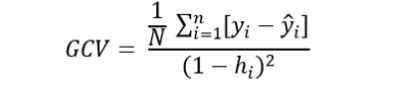



where *N* is the sample size, *y_i_* is the observed response variable for the *i^th^* observation, 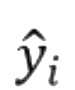 is the predicted response variable for the *i^th^* observation, and *h_i_* is the hat matrix element for the *i^th^* observation.

Finally, the scale estimate is a measure of the residual variance in the model. It represents the SD of the residuals from the model and is used to assess the model’s goodness of fit. The formula is as follows:



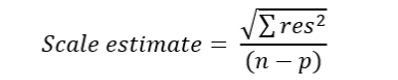



where *n* is the sample size, *p* is the number of predictors in the model, and *res* are the residual differences between the observed response variable and the predicted response variable.

A kernel estimator was used to evaluate the spatial density of the events. This technique is a nonparametric method that can estimate the probability density function (PDF) of a random variable based on observed data. In the case of CLDD reports, the PDF represents the distribution of cases across a population, which is an important factor in understanding the spread of the disease and identifying high-risk areas. The formula is defined as:



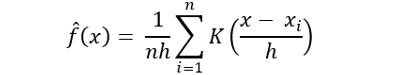



where *x* is the point at which the estimate is being made, *x_i_* represents the *i^th^* observation in the data set, *n* is the number of observations in the data set, *h* is the bandwidth parameter, and *K* is the kernel function.

In this study, to safeguard participant privacy and avoid disclosing sensitive information, we used the coordinates of CBCCs. Notably, CBCCs cater to multiple villages that are usually close to the facility. The households included in this study had at least 1 child attending the CBCC. Consequently, given the dynamic social landscape within these villages, the CBCC locations served as a suitable proxy for the frequently visited vicinities by households. These measures ensured participant confidentiality while allowing for accurate spatial analysis within the study's scope.

## Results

**Figure 2:**
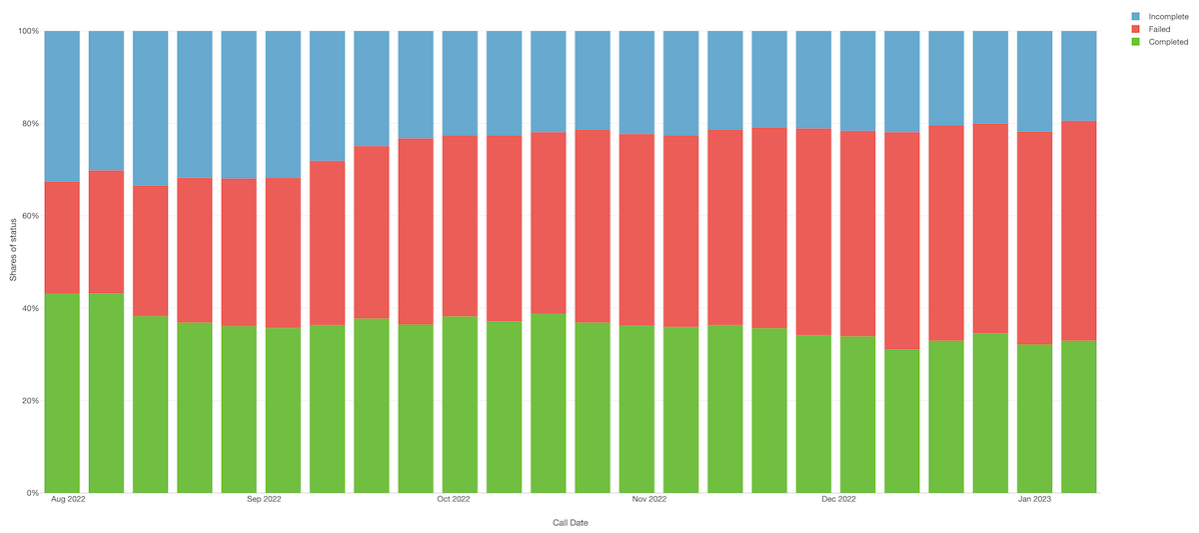
Participation profile rate according to the status of the interactive voice response (IVR) call aggregated.

**Figure 3:**
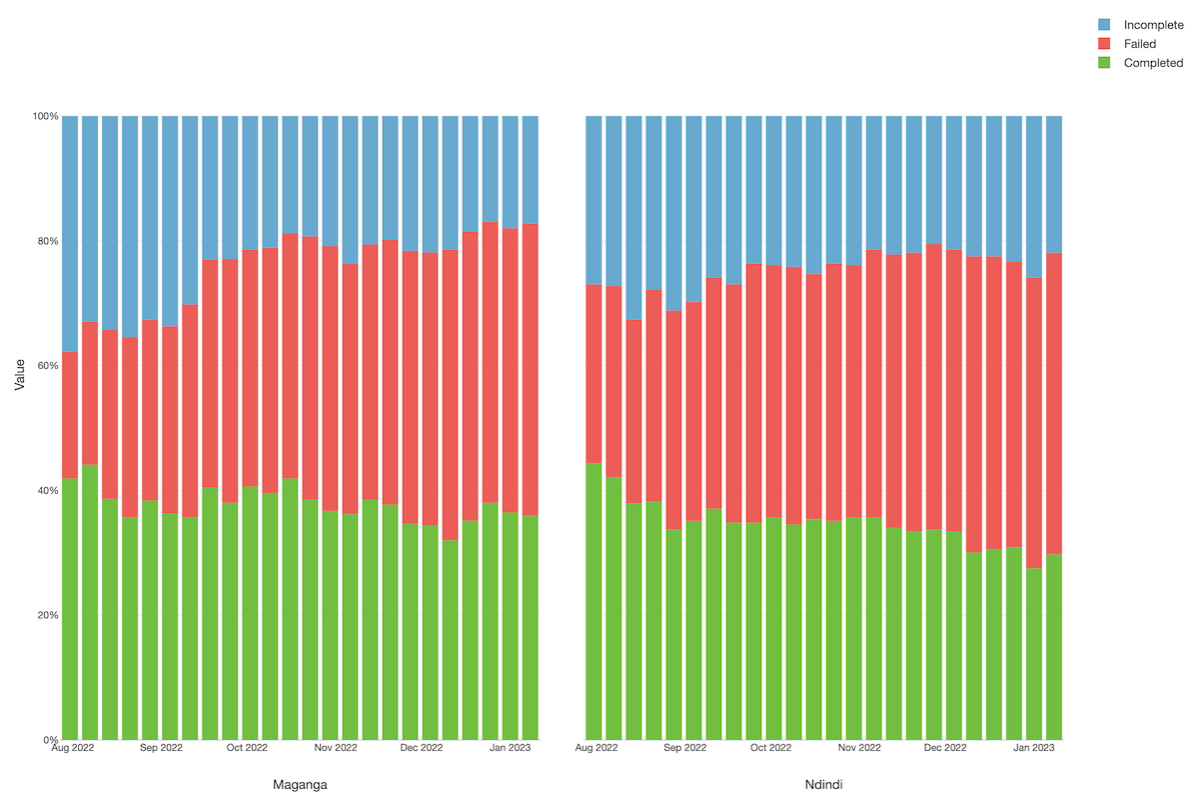
Participation profile rate according to the status of the interactive voice response (IVR) call disaggregated by Traditional Authority (TA) areas.

During the study period, our data comprised 16,280 observations, achieving a weekly participation rate of 35%. Maganga TA showed the highest average of completed calls, at 144.83 (SD 10.587), while Ndindi TA showed an average of 123.66 (SD 13.176) completed calls. The participation rates were slightly higher at the beginning of the study and declined over time, with no significant drop, possibly influenced by the sensitization efforts implemented at the CBCC level. Figure 2 displays the participation rates for each week throughout the study period, while Figure 3 presents these data disaggregated by TA area.

**Figure 4:**
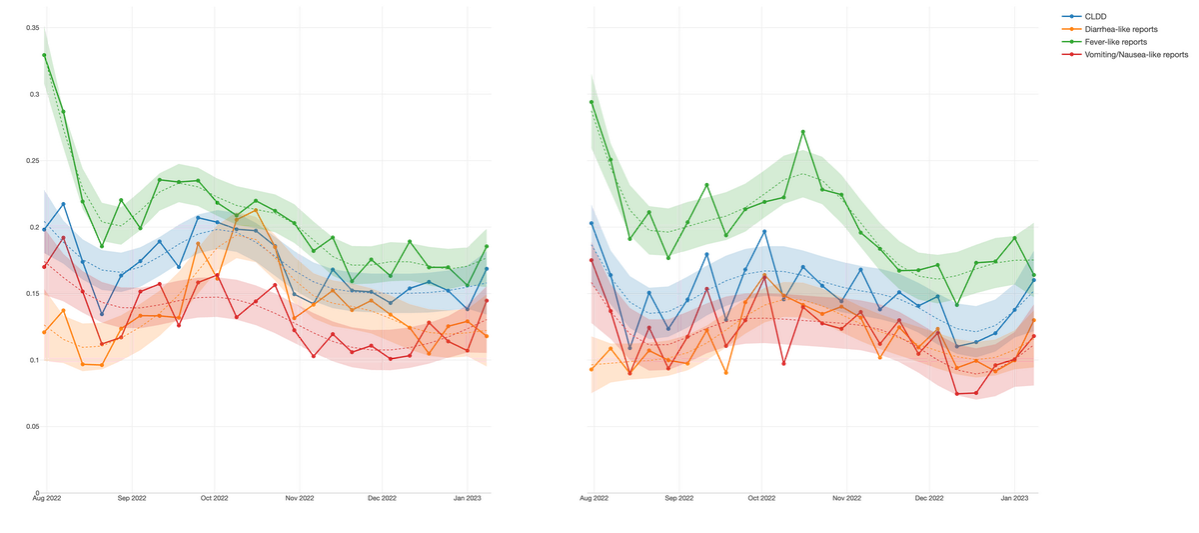
Time-series visualization of the signals, including the trend line (polynomial generalized additive model, GAM) of cholera-like diarrheal disease (CLDD), diarrhea-like reports, fever-like reports, and vomiting/nausea-like reports for Maganga Traditional Authority (TA) and Ndindi TA. The y-axis shows the percentage of reports that answered positively for the symptoms or syndrome.

Regarding the attack rates for CLDD, we found similar rates between Maganga TA and Ndindi TA, at 16% and 15%, respectively (Table 3). While fever-like reports could be taken as more sensitive than diarrhea-like reports and vomiting/nausea-like reports, using the proxy for CLDD might have helped to envelop the sensitivity of the signal, decreasing the potential number of false positives Figure 4.

**Figure 5:**
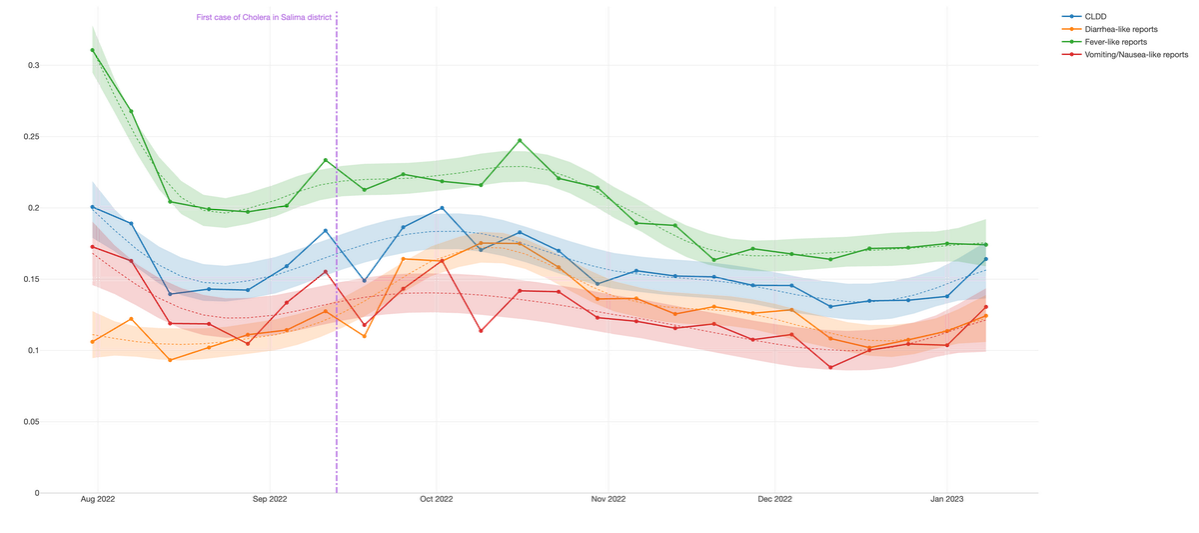
Time-series visualization of the signals, including the trend line polynomial generalized additive model, (GAM) of cholera-like diarrheal disease (CLDD), diarrhea-like reports, fever-like reports, and vomiting/nausea-like reports aggregated by Salima District. It also includes the date of the first cholera case reported in the district. The y-axis shows the percentage of reports that answered positively for the symptoms or syndrome.

Considering the aggregated view, CLDD showed a consistent signal over time (*R*^2^=0.681404) and was even stronger when isolating diarrhea-like symptoms and fever-like symptoms, as shown in Table 3. When breaking down by TA area, Maganga (Table 4) demonstrates a better performance than Ndindi (Table 5). At the beginning of the cohort, the highest activity in the time-series distribution could be explained by the initial efforts and participants’ fresh recall of the project. After 5 weeks of reports, the signal began increasing again, which might be related to the occurrence of the cholera outbreaks that hit that region during those months. The first report of cholera case in the Salima District was found on September 14, 2022, 2 days after all signals showed a spike (Figure 5). Although official reports were scarce, a hyperlocal media website confirmed the first cholera case in the Salima District [41].

**Table 3:** Attack rates at the household level based on the signals reported during the study.

Location	Signal			
	Diarrhea-like reports	Fever-like reports	Vomiting/nausea-like reports	CLDD^a^ reports
Maganga	0.1398	0.2043	0.1327	0.1699
Ndindi	0.1203	0.2059	0.1224	0.1539

^a^CLDD: cholera-like diarrheal disease.

**Table 4:** Statistical summary for the time series of signals captured by IVR^a^ technology, considering the aggregated data for Salima District.

Signal	*R^2^*	Deviance explained	GCV^b^	Scale estimate
CLDD^c^	0.681404	0.774625	0.0624334	0.0423252
Diarrhea-like reports	0.831161	0.885469	0.0422496	0.0274658
Fever-like reports	0.92951	0.954495	0.0420778	0.0260313
Vomiting/nausea-like reports	0.587607	0.692355	0.081265	0.0580976

^a^IVR: interactive voice response.

^b^GCV: generalized cross-validation.

^a^CLDD: cholera-like diarrheal disease.

**Table 5:** Statistical summary of GAM^a^ for the time series of the signals captured, considering the disaggregated data for Maganga TA^b^ and Ndindi TA areas.

TA area and signal			*R^2^*		Deviance explained		GCV^c^		Scale estimate
**Maganga**									
	CLDD^d^	0.596143		0.69602		0.020795		0.015	
	Diarrhea-like reports	0.711076		0.796532		0.0256		0.017277	
	Fever-like reports	0.899293		0.9338		0.016462		0.0103705	
	Vomiting/nausea-like reports	0.537684		0.632479		0.0247917		0.0188871	
**Ndindi**									
	CLDD	0.421066		0.576651		0.0414793		0.0290681	
	Diarrhea-like reports	0.711076		0.796532		0.0256		0.017277	
	Fever-like reports	0.785284		0.851777		0.0343511		0.0227253	
	Vomiting/nausea-like reports	0.408624		0.560045		0.047594		0.0304854	

^a^GAM: generalized additive model.

^b^TA: Traditional Authority.

^c^GCV: generalized cross-validation.

^d^CLDD: cholera-like diarrheal disease.

**Figure 6:**
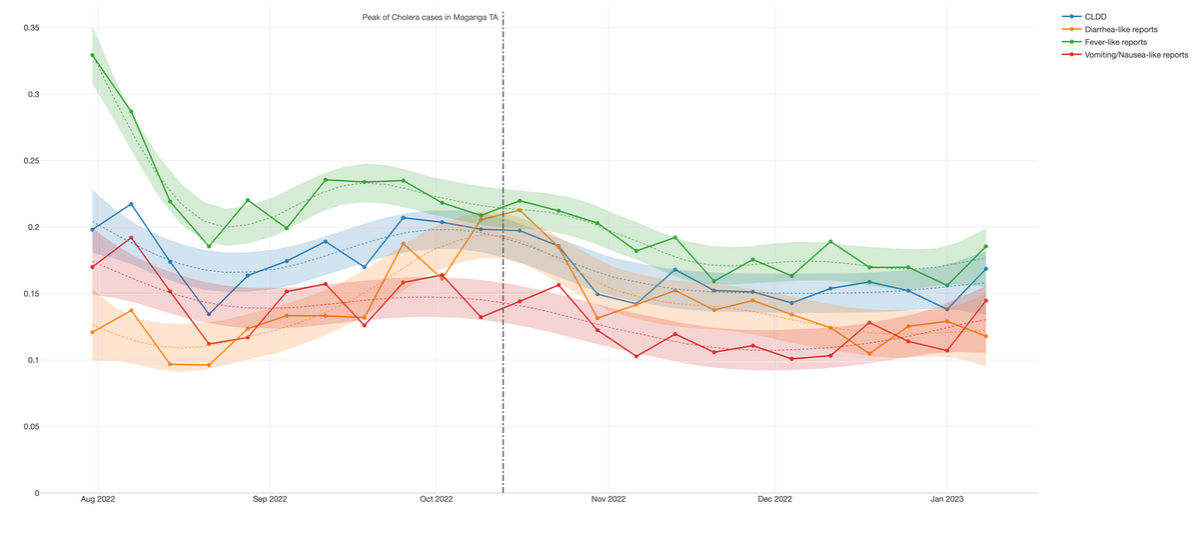
Time-series visualization of the signals, including the trend line (polynomial generalized additive model, GAM) of cholera-like diarrheal disease (CLDD), diarrhea-like reports, fever-like reports, and vomiting/nausea-like reports for Maganga Traditional Authority (TA). It also includes the peak of cholera cases in that TA area. The y-axis shows the percentage of reports that answered positively for the symptoms or syndrome.

In the subsequent weeks, the peak of cholera cases in the Maganga TA area was detected, where an ascending pattern in the diarrhea-like reports could be observed (Figure 6; [42]). Three weeks before this peak, CLDD reports were at their highest level when this area was considered in isolation.

**Figure 7:**
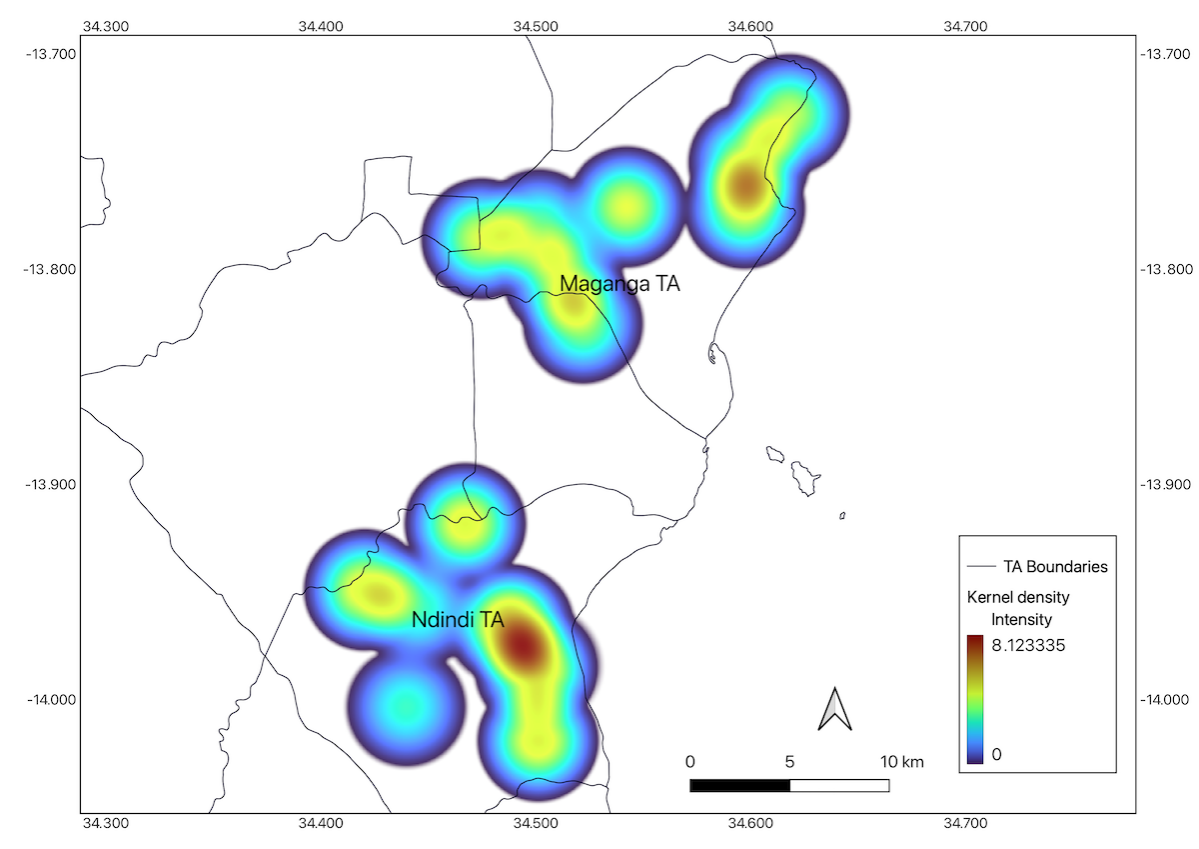
Kernel density estimator for the cholera-like diarrheal disease (CLDD) reports in both Maganga Traditional Authority (TA) and Ndindi TA areas aggregating the whole study period.

Regarding the spatial distribution of the CLDD reports, even assuming a homogeneous distribution of these events over the regions, there is a notable concentration near the lake vicinity (Figure 7). The Salima District Council locally issued a “Cautionary Statement on Cholera” on October 13, 2022, aiming to provide the populace with guidance. In this statement, they reported: “Salima District has registered a total of 54 cases as of 13 October 2022 with TA Maganga along the lake shore being the most affected, with 45 cases” [43].

This observation corroborates the proxies’ ability to identify trends indicative of a possible cholera outbreak in high-risk areas.

## Discussion

### Principal Findings

In this study, we implemented a PS system using IVR in 2 districts of Malawi and assessed its feasibility, acceptability, and effectiveness in detecting early signals of CLDD outbreaks. During the 24-week follow-up period, the system captured 16,280 observations, and the average weekly participation rate was 35%, indicating the strategy’s feasibility and acceptability according to the literature [24,44-46].

In summary, our findings demonstrate that this method might be effective in identifying CLDD with a notable and consistent signal being captured over time (*R*^2^ =0.681404). The signal showed a significant increase coinciding with cholera outbreaks in the region. This pattern was notably observed with the first cholera case in the Salima District, detected shortly after a spike in our CLDD data (Figure 5). Despite limited official reporting, this outbreak was confirmed through hyperlocal media sources. Furthermore, a subsequent analysis highlighted a peak in cholera cases in the Maganga TA area, preceded by a rise in CLDD reports of diarrhea-like symptoms underscoring the potential of CLDD in early outbreak detection and response facilitation. The local media released a breaking news report on October 15, 2022, highlighting the “Cautionary Statement on Cholera” that the Salima District Council issued on October 13, 2022. Signed by the Director of Health and Social Services, the statement aimed to provide guidance to the population. It mentioned a total of 54 cholera cases in Salima, emphasizing that TA Maganga accounted for 45 cases. The statement also underscored that the regions most affected were those in close proximity to the lake [43].

Traditional surveillance systems can have limitations in detecting early signals of outbreaks, particularly in resource-limited settings [7,10,14]. Therefore, PS has been proposed as an additional approach that leverages the local knowledge and resources of communities to identify early outbreak signals.

The successful implementation of PS must consider the site's geolocation, epidemiological profile, seasonality, and available telecommunication infrastructure [47]. In this study, Maganga and Ndindi encompass a vast territorial area bordered by Malawi Lake, the ninth-largest freshwater lake on Earth [48,49]. The lake plays a vital role in the region's economy, demographic density, and definition of 2 different seasons—the dry and wet seasons [48]. Thus, during the wet season between December and April, the population expects floods followed by disruptions in the WASH infrastructure, and consequently, an increase in overall diarrhea cases [16,48].

Moreover, in 2019, there were more than 5 million mobile subscriptions, with a gradual increase in mobile penetration over the years, predominantly through prepaid subscriptions [50]. The growth in mobile penetration is evident, with reports indicating approximately 12.27 million mobile subscribers at the beginning of 2022 [51]. Regarding internet coverage, the International Telecommunication Union showed that only 10% of the Malawi population was using the internet until 2019 [49]. Although internet penetration is growing, reports from 2022 showed that almost 80% of the country’s population remained offline, with internet access being particularly low in areas away from cities [52]. Bearing that in mind, models of PS conducted through mobile apps, such as Flu Near You, AfyaData, or the Guardians of Health platform, would likely not achieve significant engagement in the most remote areas of Malawi. Therefore, the choice of a self-report survey via IVR may be the most appropriate strategy for this setting [33,40,44,53,54].

PS strategies have been applied worldwide with positive results in different settings and events, successfully tracing pandemics such as Zika, H1N1, and COVID-19. PS has the potential to anticipate outbreaks and provide a quicker overview, guiding authorities to the hotspots of specific diseases [25,53-55]. Despite limitations in the field of technology and communication, PS strategies can rely on affordable systems that have been successfully implemented in underresourced areas, such as Malawi [26]. By leveraging the IVR design, it is possible to establish a less expensive, flexible, scalable, and reliable system that captures data voluntarily and provides information that is not possible to capture using traditional surveillance methods. This approach can empower communities to take an active role in anticipating disease outbreaks, even in settings where internet coverage is limited [34,56,57].

### Study Limitations

Our study has several limitations. In terms of study design, population displacement and changes in the household configuration affected the consistency of the individuals being reported using the same household phone number. Constant phone number changes resulted in us losing contact with an entire household. Pitfalls in the adherence rate over the weeks could occur due to recipients perceiving the repeated calls with identical questions as spam, potentially leading them to avoid answering that specific phone number.

Selection bias is another significant limitation. Not all individuals may have access to a mobile phone, and those who have may differ in socioeconomic status, education level, or other factors that may affect their willingness to participate in the study. This could result in an unrepresentative sample and affect the generalizability of the results.

Another limitation is that the quality and accuracy of the data collected through IVR may be affected by factors such as poor network coverage, low battery, or other technical issues that may prevent individuals from completing the survey or lead to missing data. Additionally, individuals may not always report their symptoms accurately or truthfully, which could affect the validity of the data collected through IVR. Validating PS data with traditional sources in low-income settings is also challenging due to the scarcity of disease surveillance data. In many low-income settings, disease surveillance systems may be underresourced or underdeveloped, resulting in delays in the collection, analysis, and reporting of disease data. Consequently, it can be challenging to validate the accuracy of the data collected through PS with traditional sources. This sum of constraints can lead to a lack of confidence in the accuracy of the PS data and limit its usefulness for public health decision-making. Moreover, traditional sources of disease data, such as hospital records or laboratory test results, may not always be available or accessible in low-income settings, further complicating the validation process. Therefore, while PS has the potential to provide valuable data on disease trends and outbreaks in low-income settings, its usefulness and advantages may be limited by the lack of timely and reliable validation data from traditional sources.

### Conclusion

IVR systems have the potential to facilitate PS in low-income and low-resource countries by enabling efficient and cost-effective data collection. In this study, weekly automated phone calls were made to a representative sample of the population over a 6-month period, during which participants answered a consistent set of 10 questions related to cholera and malaria and associated risk factors. In low-income countries and settings with limited technological resources, PS using this method can be particularly useful in preventing CLDD outbreaks for several reasons. First, IVR allows for the timely detection of cases by rapidly identifying suspected CLDD cases within communities, providing public health officials with real-time data to respond promptly and contain potential outbreaks. Second, by using a standardized set of questions, the IVR system ensures that data collected across different participants and time points are consistent and comparable, thus improving the surveillance system’s reliability. Additionally, the use of phone-based surveys enables data collection from geographically dispersed and hard-to-reach populations, overcoming logistical barriers typically encountered in low-resource settings. Finally, by longitudinally monitoring the same set of participants over 6 months, the IVR-based PS system can capture temporal trends and identify emerging risk factors, enabling targeted and context-specific interventions to prevent and control disease outbreaks.

## Abbreviations

CBCC: community-based childcare center
CFR: case-fatality ratio
CLDD: cholera-like diarrheal disease
COMREC: College of Medicine Research and Ethics from Malawi
ETL: extract, transform, and load
GAM: generalized additive model
GCV: generalized cross-validation
IVR: interactive voice response
KUHES: Kamuzu University of Health Sciences
PDF: probability density function
PS: participatory surveillance
TA: Traditional Authority
WASH: water, sanitation, and hygiene
WHO: World Health Organization

## Data Availability

Data related to this study are available upon request. Interested parties can contact the corresponding author to access the data sets, which are shared in accordance with applicable privacy and ethical guidelines.
